# Wild *Vitex agnus-castus* L.: Phytochemical Characterization, Acute Toxicity, and Bioactive Properties

**DOI:** 10.3390/molecules28135096

**Published:** 2023-06-29

**Authors:** Mohamed Ali Boujbiha, Hassiba Chahdoura, Khaoula Adouni, Borhane Eddine Cherif Ziani, Mejdi Snoussi, Yasmine Chakroun, María Ciudad-Mulero, Virginia Fernández-Ruiz, Lotfi Achour, Boulbaba Selmi, Patricia Morales, Guido Flamini, Habib Mosbah

**Affiliations:** 1 Laboratory of Bioresources: Integrative Biology and Exploiting, Higher Institute of Biotechnology of Monastir, University of Monastir, Avenue Taher Hadded BP 74, Monastir 5000, Tunisia; boujbihamohamedali@gmail.com (M.A.B.); khawla.adouni01@gmail.com (K.A.); yasminechakroun9@gmail.com (Y.C.); lotfiachour@yahoo.fr (L.A.); selmi_boulbaba@yahoo.fr (B.S.); mosbah_habib@yahoo.fr (H.M.); 2 Unité de Recherche “Génomique, Biotechnologie et Stratégies Antivirales”, Institut Supérieur de Biotechnologie, Université de Monastir, BP74, Avenue Tahar Hadded, Monastir 5000, Tunisia; hassiba_chahdoura@yahoo.fr; 3 Centre de Recherche Scientifique et Technique en Analyses Physico-Chimiques CRAPC, Tipaza 42000, Algeria; ziani.ensa@gmail.com; 4 Department of Biology, University of Hail, Ha’il P.O. Box 81451, Saudi Arabia; snmejdi@yahoo.fr; 5 Laboratory of Genetics, Biodiversity and Valorisation of Bioresources, High Institute of Biotechnology-University of Monastir, Monastir 5000, Tunisia; 6 Department of Nutrition and Food Science, Faculty of Pharmacy, Complutense University of Madrid, Plaza Ramon y Cajal, s/n, E-28040 Madrid, Spain; vfernand@ucm.es; 7 Dipartimento di Farmacia, Università di Pisa, Via Bonanno 6, 56126 Pisa, Italy; guido.flamini@unipi.it; 8 Centro Interdipartimentale di Ricerca “Nutraceutica e Alimentazione per la Salute”, Università di Pisa, Via del Borghetto 80, 56124 Pisa, Italy

**Keywords:** *Vitex agnus-castus* L., HS-SPME/GC-MS, antioxidant capacity, acute toxicity, anti-inflammatory, analgesic, gastroprotective potential

## Abstract

Wild *Vitex agnus-castus* (VAC) is a Mediterranean plant that is rich in bioactive metabolites. This study aimed to validate, for the first time, the beneficial use of VAC fruits and fruit decoctions (VFDs) through in vitro and in vivo trials. Forty-one volatile components were detected in VAC fruits, with 1,8-cineole (30.3%) comprising the majority. The antioxidant activity of VFD was measured by using different in vitro methods (EC_50_ of 0.16 mg/mL by β-carotene bleaching inhibition assay) and by measuring the DNA protection power. Using the disc diffusion assay, the antimicrobial activity of VFD was evaluated, and it exhibited a noticeable anticandidal activity. VFD did not cause any toxicity or mortality in rats treated with doses > 200 mg/kg. Using the acetic acid writhing test, the antinociceptive activity of VFD was measured. Our results showed that VFD at 200 mg/kg exhibited a higher analgesic activity (81.68%) than acetylsalicylic acid used as a positive control (74.35%). Its gastroprotective ability was assessed by HCl/ethanol-induced gastric lesions, which were remarkably inhibited (84.62%) by intraperitoneal administration of VFD. This work helps to validate the popular use of VAC to treat nociceptive, inflammatory, and gastric disorders and encourages researchers to further investigate the identification of pharmacological compounds from this species.

## 1. Introduction

Wild food plants have been used for medicinal purposes in the treatment of several diseases for a long time in human history. Thousands of wild plant species worldwide exhibit medicinal properties and are still in use as part of alternative medicine. They are also important sources of many bioactive compounds that can be employed in certain therapies due to their analgesic and anti-inflammatory properties, among others [1,2,3]. On one hand, pain is a psychological, physical, and emotional sensation, which can be associated with tissue damage. It has a negative impact that significantly affects the daily lives of individuals worldwide. On the other hand, inflammation is an important global concern due to its incapacitating symptoms, resulting in extensive suffering and lost productivity. Nowadays, there is an increased interest from pharmaceutical and scientific sectors in the use of medicinal plants as analgesics, and there is a crucial need for new anti-inflammatory drugs, especially those derived from natural products [4]. Due to the confirmed side and toxic effects of many synthetic drugs, in recent years, a trend has been observed to explore natural resources as interesting alternatives [5]. Nature has always been and is still recognized as the oldest and the richest reservoir of the most comprehensive pharmacy. The chief reason for the interest towards herbal medicines is that many people consider that the use of these alternative medicines has no side effects, is cost effective, and is much safer [6]. Additionally, experimental studies are necessary for the validation of ethnopharmacological knowledge regarding herbal medicine. Indeed, phytochemical and pharmacognostic evaluations are the required steps to check the safety and the efficiency of the use of medicinal plants. Thus, many herbs are medicinal plants, which are always available and easy to use, but many people are unaware of their amazing properties. In this context, it is important to preserve a high degree of traditional knowledge related to wild medicinal plant use. However, modernization is gradually displacing traditional knowledge. Most medicinal plants belong to *Rosaceae*, *Asteraceae*, and *Lamiaceae* families, and infusion and decoction have been the most preferred preparations for using these plants in folk medicine [2,3]. Among these beneficial plants, *Vitex agnus-castus* L. (VAC) was used by practitioners of phytotherapy to treat several ailments and is still the chief alternative for most people worldwide [7,8,9,10].

The *Vitex* genus belongs to the *Lamiaceae* family, which comprises 750 taxa distributed all over the world [10,11,12]. The safety and effectiveness of various *Vitex* taxa have been reported in previous pharmacological studies, which have made this genus a popular candidate in phytochemical and ethnobotanical research [13]. Among these taxa, *Vitex agnus-castus* (VAC) grows widely on the riverbanks and on the shores of the Mediterranean region, central Asia, and Southern Europe [14]. All the organs of the herb are medicinally of interest [7] and have a long history (over 2000 years) of use since ancient Greek and Roman times. The most used part of the plant is the ripe dried fruit in the form of extracts/concentrates [8]. Based on the ethnomedicine of several nations, VAC is among the main plants used for relieving several female disorders, such as spasmodic dysmenorrhea and menstrual pain. In popular medicine, VAC has been also used in the treatment of eye diseases, acne, snake bites, scorpion stings, pain, rheumatism, swelling, and stomachaches, and also as an anti-inflammatory and emmenagogue agent [10,15,16]. Scientific reviews are available and they have endorsed the efficacy of the use of VAC and reported its analgesic, anti-inflammatory [17], antimicrobial [18], diuretic, and digestive properties [19]. These therapeutic effects have been attributed to certain bioactive compounds isolated from VAC fruits, such as phenolic acids and their derivatives, flavonoids, tannins, iridoids, diterpenoids, volatile oils (limonene, pinene, and sabinene), and essential fatty acids (oleic, linolenic, palmitic, and stearic acids) [20]. It was suggested that these potential phytochemicals, which confer the antioxidant activity to the VAC fruits [21], could provide bioactive mechanisms in the treatment and the prevention of many diseases associated with oxidative stress, such as diabetes mellitus, cancer, neurodegenerative, and cardiovascular disorders. Moreover, the use of extracts with non-particular side effects has become one of the most popular alternative therapies for treating such ailments. However, VAC extracts are associated with some soft and infrequent side effects, including skin problems, headaches, diarrhea, vertigo, and palpitations, which disappear after stopping its use [9,22,23]. On the other hand, there are no reports of herb–drug interactions involving VAC.

VAC, locally known as “Kaf Maryem” in Tunisia, is one of the available species used in traditional medicine. To the best of our knowledge, there are no specific phytochemical studies of Tunisian VAC fruits for their in vivo analgesic, anti-inflammatory, and anti-ulcer activities. Thus, the aim of this study was to validate the use of VAC in Tunisian folk medicine. Therefore, for the first time, a phytochemical analysis was conducted and the antimicrobial activity, acute toxicity, and analgesic, anti-inflammatory, and anti-ulcer potential of the VFD (Vitex fruits decoction) were evaluated.

## 2. Results

### 2.1. Phytochemical Compounds

The dietary fiber, mineral composition, and soluble sugars of Tunisian *Vitex agnus-castus* fruits were analyzed, and the results are shown in Table 1. Dietary fiber was the major macronutrient detected in VAC fruits (85.02 g/100 g dw), and insoluble dietary fiber was the main fraction.

Regarding the mineral composition of *V. agnus-castus* fruit, potassium and calcium were the major macroelements found, with values of 1428.84 and 781.29 mg/100 g (dw), respectively. Among the analyzed microelements, iron and zinc were the most abundant (5.89 and 4.69 mg/100 g fw, respectively) in *V. agnus-castus* fruits, while copper and manganese were detected in trace amounts (1.9 and 0.44 mg/100 g fw, respectively).

The content of soluble sugars appears in Table 1. It was observed that these compounds were found in low concentrations, with sorbitol and arabinose being the majority, with values of 470.40 and 470.08 mg per 100 g (dw), respectively. To the authors’ knowledge, this is the first work which studies the soluble sugar composition of *V. agnus-castus* fruits.

In the present study, a total of 41 headspace volatile compounds sampled by SPME were characterized in Tunisian fruits of *V. agnus-castus* by GC–MS. The identified compounds, as well as their percentages and their retention indices, are summarized in Table 2. As indicated in this table, 99.7% of the total volatile compounds was detected and distributed in five chemical classes: sesquiterpene hydrocarbons (45.9%), oxygenated monoterpenes (34.5%), monoterpene hydrocarbons (17.9%), oxygenated sesquiterpenes (1.3%), and non-terpene derivatives (0.1%). Among the 41 detected compounds, 1,8-cineole (30.3%), germacrene *D* (11.8%), (*E*)-β-farnesene (9.4%), β-caryophyllene (9.1%), sabinene (7%), and α-pinene (6.2%) were the major ones. 

In this study, the total phenolic content of VFD (*V. agnus-castus* fruit decoction) was measured by using the Folin–Ciocalteu method, and the results (78.53 mg/g extract) are shown Table 3. The flavonoid amount in VFD was also evaluated (Table 3) and the obtained value was 56.52 mg/g extract. For further characterization, the flavonol and ortho-benzenediol contents of VFD were also measured for the first time, resulting in values of 33.7 mg RE/g and 30.47 mg HE/g, respectively (Table 3).

### 2.2. Antioxidant Activity

**Figure 1:**
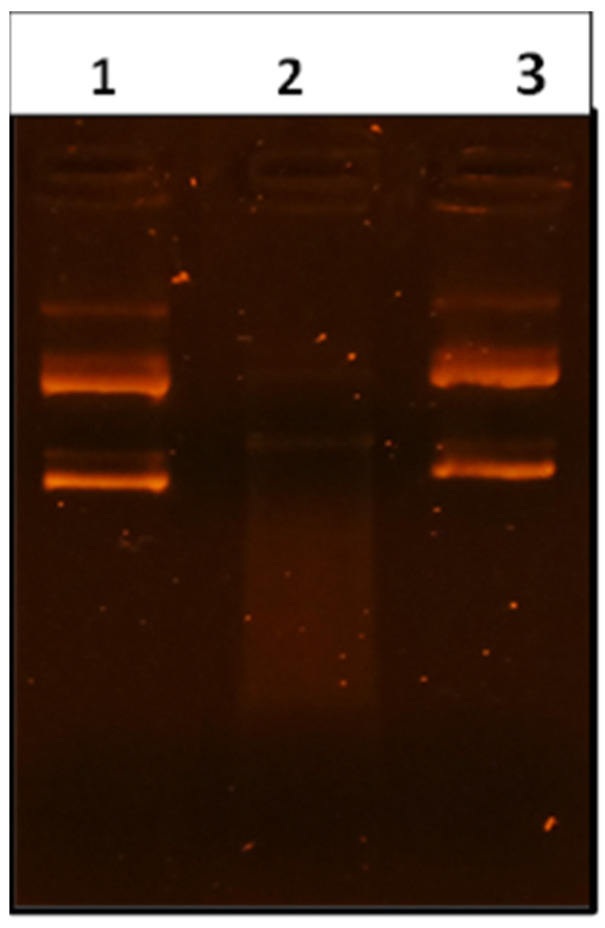
Gel electrophoresis conformation of the pGEM^®^- (Promega Biotech Ibérica S.L. Madrid, Spain) T plasmid incubated with Fenton’s reagent in the presence or absence of VFD. Lane 1: native pGEM^®^-T DNA (0.5 µg); Lane 2: DNA treated by Fenton’s reagent; lanes 3: DNA + Fenton’s reagent + VFD (2 mg/mL).

The antioxidant capacity of VFD (*V. agnus-castus* fruit decoction) was evaluated by checking the EC_50_ using six different methods (Table 3), namely DPPH (EC_50_ = 0.64 mg/mL), FRAP (EC_50_ = 0.35 mg/mL), ABTS (EC_50_ = 1.03 mg/mL), β-carotene bleaching (EC_50_ = 0.16 mg/mL), metal chelating (EC_50_ = 0.44 mg/mL), and TBARS (EC_50_ = 3.108 mg/mL). In addition to these spectrophotometric methods, the DNA protection power of VFD was also evaluated. Pre-incubation of DNA with 2 mg/mL of VFD protected the plasmid DNA against Fenton’s reagent damage, with a higher preservation of the band intensity of supercoiled DNA (Lane 3, Figure 1). Therefore, we suggest that the reduction (or inhibition) in DNA damage may be due to the ferrous ion chelating power of VFD, which has been also shown by the measure of its antioxidant activity by the ferrous chelating method (Table 3). 

### 2.3. Antimicrobial Activity

The antibacterial/antifungal activities of VFD grown in Tunisia are expressed as diameters of the inhibition zone (IZ) (Table 4). The antimicrobial effects of VFD were determined by the disc diffusion and microdilution methods. As it is shown in Table 4, our extract exhibited a moderate activity against all the tested bacterial strains. In contrast, VFD was very effective versus all the yeast strains, with various diameters of the inhibition zone (Table 4) when compared with the standard drug (Amphotericin B). The IZ ranged from 8.66 ± 0.66 to 15.66 ± 0.23 mm.

The MBC/MIC and MFC/MIC ratios determine the antibacterial and antifungal power of VFD. When these ratios are ≤4, the extract is considered as a bactericide or a fungicide. When it is >4, the extract is described as bacteriostatic or fungistatic. Therefore, the results included in Table 4 show that VFD is a fungicide agent.

### 2.4. Acute Toxicity Study

During the study period (14 days), the tested doses of VFD revealed no toxic effects, no changes in general behavior (salivation, tremors, and diarrhea), and no mortality of treated rats. This suggests that the LD_50_ value of VFD could be much higher than 500 mg/kg and that VFD could be considered safe for acute ingestion. 

Furthermore, the blood chemical parameter (urea, creatinine, AST, ALT, and CRP) data (Table 5) suggested the non-toxicity of our extracts, as no significant differences (*p* > 0.05) were evident between treated and control animals. Moreover, it is noteworthy that the administration of VFD had no adverse effect on liver or kidney functions.

### 2.5. Analgesic Activity

The analgesic effect of VFD was evaluated through pain induced by acetic acid injected intraperitoneally in experimental mice [24]. When compared to the untreated group (negative control), the group administrated with ASL (200 mg/kg) exhibited a significant (*p* < 0.001) decrease in the number of writhes. The analgesic activity of VFD was statistically significant (*p* < 0.001) in a dose-dependent manner (Table 6). Moreover, the administration of VFD at a dose of 200 mg/kg showed a better percentage of inhibition than that of the positive control (81.68% vs. 74.35%). 

### 2.6. Anti-Inflammatory Activity

As shown in Table 7, and compared to the control group, pretreatment of animals with VFD one hour before carrageenan injection exhibited an effective anti-inflammatory activity in a dose- and time-dependent manner. VFD at 200 mg/kg showed a more efficient anti-inflammatory effect than the standard anti-inflammatory drug (ASL) (*p* < 0.05) at the fifth hour after carrageenan injection, as reflected by a significant decrease in paw volume (40.49%). 

### 2.7. Gastroprotective Activity

**Figure 2:**
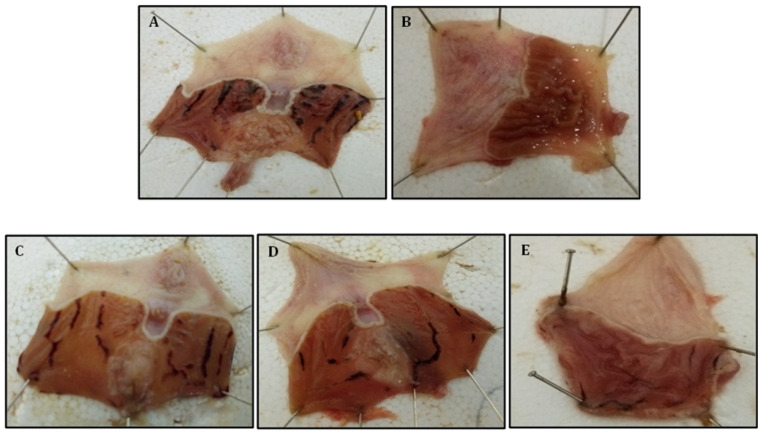
Macroscopic appearance of gastric lesions in rats. (**A**) The stomach of control rats, (**B**) the stomach of rats treated with omeprazole (30 mg/kg), (**C**) the stomach of rats treated with VFD (50 mg/kg), (**D**) the stomach of rats treated with VFD (100 mg/kg), and (**E**) the stomach of rats treated with VFD (200 mg/kg).

The gastroprotective properties of the VFD were highlighted by measuring the ulcer index. As shown in Figure 2, HCl/EtOH administered to rats by oral gavage caused severe stomach mucosal alteration with various sizes of hemorrhagic bands. The best protection of the mucosa (94.46%) was recorded when using omeprazole as a reference drug (30 mg/kg). However, intraperitoneal administration of VFD (50, 100, and 200 mg/kg) also showed a dose-dependent protective activity on HCl/EtOH-induced gastric ulcers (Figure 2).

Our finding showed that pre-treatment with VFD decreased the degree of gastric mucosal alterations compared to the control group. At 200 mg/kg, VFD gives an inhibition percentage of 84.62%, which is near to that of omeprazole (Table 8). 

## 3. Discussion

### 3.1. Phytochemical Compounds

Comparing the obtained results with those found in the scientific literature, it was noted that the Tunisian *V. agnus-castus* fruits were richer in dietary fiber compared to *Vitex payos* (Lour.) Merr. fruits (60 ± 0.4 g/100 g dw) [25]. Thus, the inclusion of *V. agnus-castus* in human diets could be very interesting as these fruits are high in dietary fiber according to European Regulation (EC) No 1924/2006, since they provide more than 6 g of fiber per 100 g [26]. Regarding the mineral composition, it should be noted that the detected calcium rate in VFD was significantly higher than that measured in the fruits of *Vitex payos* (Lour.) Merr. collected in Kenya [25]. The mineral composition of the fruit pulp of *Vitex payos* (Lour.) Merr. has been studied by other authors [25], who reported values of iron (11.9 mg/100 g dw) and manganese (3.9 mg/100 g dw) higher than those showed in Table 1. However, the values of zinc (1.9 mg/100 g dw) reported by the cited authors were lower than those obtained in our study (4.69 mg/100 g dw).

As it has been previously stated, in the present study, a total of 41 headspace volatile compounds sampled by SPME were characterized in Tunisian fruits of *V. agnus-castus* by GC–MS. Solid-phase microextraction (SPME) is a technique used to characterize volatile compounds in aromatic plants without generation of thermal changes, as occurs in the steam-distillation process that is applied to obtain essential oils. For this reason, a direct comparison between SPME and essential oil analysis results is not possible. However, according to the literature, the same major constituents were previously detected in Turkish VAC volatile oils [21]. These authors identified 27 compounds, with 1,8-cineole (24.98%), sabinene (13.45%), α-pinene (10.60%), α-terpinyl acetate (6.66%), and *(Z)*-β-farnesene (5.40%) as the major ones [21]. Likewise, the same main constituents were detected in the volatile fraction of *V. agnus-castus* leaves gathered in Iran [27]. It should be noted that although the major compounds are almost the same, there are significant differences in their relative amounts and this can be related to maturity, the method of extraction, or the distillation period [28].

Phenolic compounds are frequently isolated from various fruits, vegetables, and herbs where they provide a defense against oxidative stress from reactive oxygen species because of their chemical structure [29,30,31,32]. Polyphenols are also known for their ability to prevent the oxidation of fatty acids, and thereafter provide additional value to plants used as food ingredients [33]. As it has been indicated above, in this study, the total phenolic content of VFD (*V. agnus-castus* fruit decoction) was measured by using the Folin–Ciocalteu method, and the obtained concentration (78.53 mg/g extract) was less than that recorded for Turkish fruit aqueous extracts (112.46 mg GAE/g extract) [34] and seven times higher than that reported by other authors for the leaves aqueous extract of *V. agnus castus* growing in Morocco [35]. The flavonoid amount in VFD (56.52 mg/g extract) determined in our study was almost three times higher than the amount reported for Turkish *V. agnus-castus* aqueous fruit extracts [21]. In the present work, the flavonol and ortho-benzenediol contents of VFD were also measured for the first time.

### 3.2. Antioxidant Activity

Antioxidants play a pivotal role in oxidative stress prevention, which is responsible for triggering several disorders, including diabetes, cardiovascular diseases, atherosclerosis, neurodegenerative diseases, cancer, and aging [36]. According to the literature, several methods have been applied to evaluate the antioxidant effect of natural compounds, including polyphenols, flavonoids, and volatile compounds. As it has been previously indicated, in our case, the antioxidant capacity of VFD (*V. agnus-castus* fruit decoction) was evaluated by checking the EC_50_ using six different methods, namely DPPH, FRAP, ABTS, β-carotene bleaching, metal chelating, and TBARS. Comparing the obtained results with those found in the scientific literature, it is observed that similar EC_50_ value was obtained by a DPPH assay for the aqueous extracts of the fruits of *V. agnus-castus* cultivated in Turkey [21]. In same way, it has been shown that the leaf extracts of *V. agnus-castus* collected from Morocco exhibit an important antioxidant activity (EC_50_ = 1.01 ± 0.019 mg/mL) and a very strong positive correlation with their content of phenols and flavonoids (*p* < 0.05) [35]. This strong relationship between total phenolic content and the antioxidant activity of plant extracts has been well demonstrated by several researchers [37,38]. Our findings strongly agree with these reports. In the present study, the DNA protection power of VFD was also evaluated. It could be suggested that the reduction (or inhibition) in DNA damage may be due to the ferrous ion chelating power of VFD, which has been also evidenced by its antioxidant activity by the ferrous chelating method (Table 3). These results are in line with those published by other authors who reported that the hydroethanolic extract of *Vitex negundo* leaves exhibited potential in vitro antioxidant and DNA protecting activities [39].

### 3.3. Antimicrobial Activity

The assays carried out to evaluate the antibacterial/antifungal activities of VFD grown in Tunisia provided similar results to those reported for the methanolic extract of Turkish *V. agnus-castus*, which did not exhibit antibacterial activity against all the tested bacteria [18]. On the contrary, it possessed a noticeable antifungal activity against *Candida* strains. This effective anticandidal activity of VFD was also underlined by other authors, who reported noticeable sensitivities of all the tested *Candida* strains when using Moroccan *V. agnus-castus* essential oils [40]. Recently, it has been shown that the aqueous extract of Iranian *Vitex* has antifungal activity against clinical isolates of *C. albicans* [41]. The antifungal activity of our Tunisian *V. agnus-castus* extract may be due to the occurrence of dominant compounds such as 1,8-cineole (30.3%), germacrene *D* (11.8%), (*E*)-β-farnesene (9.4%), β-caryophyllene (9.1%), sabinene (7%), and α-pinene (6.2%), which are well known for their antifungal activities [42,43].

As has been exposed above, VFD is a fungicidal agent. Accordingly, since VFD is a potential source of bioactive compounds with considerable activity against different pathogenic *Candida* species, it could be considered as a potential agent to treat nosocomial infections.

### 3.4. Acute Toxicity Study

The acute toxicity of VFD was evaluated and the obtained results were in good agreement with those previously reported by other researchers, who studied the toxicity of methanolic extracts of *V. agnus-castus* fruits collected in Iraq. These authors did not notice any significant differences in organ weight between treated and untreated animals and there was an absence of any morphological modification [44]. Additionally, it has been reported that the administration of a single oral dose of crude extracts of *V. agnus-castus* leaves growing in India was not followed by any serious abnormalities on follow-up [45].

Furthermore, the blood chemical parameters (urea, creatinine, AST, ALT, and CRP) were measured. AST and ALT, as liver function biomarkers, are leakage enzymes, whose increased blood levels reflect liver cell injury [46]. Additionally, creatinine and urea are kidney function markers [47]. Therefore, the increase in serum levels of these biomarkers is considered as an indicator of a disorder in nephron functions [48]. It is remarkable that the administration of VFD has no adverse effect on liver and kidney functions.

### 3.5. Analgesic Activity

As it has been previously indicated, the analgesic effect of VFD was evaluated through pain induced by acetic acid injected intraperitoneally in experimental mice [24]. The findings were in good agreement with those previously reported by other authors [17,27], with the essential oil of the same species cultivated in Iran and using various visceral inducing tests. Our results showed that VFD relieved pain, probably by cyclooxygenase enzyme inhibition, which thereafter generates a decrease in pain mediator production, such as prostaglandins, histamine, serotonin, and bradykinin [49], and stimulation of the ventral neurons of the spinal cord [50]. Furthermore, the abundance of 1,8-cineole (30.3%), germacrene D (11.8%), β-caryophyllene (9.1%), sabinene (7%), and α-pinene (6.2%) in VFD could explain this potent analgesic activity. Indeed, it was shown that β-caryophyllene has agonistic activity on the non-psychoactive CB2 cannabinoid receptor [51]. Moreover, it was reported that β-caryophyllene inhibits inflammation induced by dextran sulfate sodium injection in mice [52] and provides a local anesthetic effect [53]. In addition, evidence of the analgesic effect of α-pinene has been demonstrated in different pain models [54].

### 3.6. Anti-Inflammatory Activity

Subplantar carrageenan injection of *Wistar* rats to induce edema has been frequently used as a standard test to unearth new anti-inflammatory agents, as well as to elucidate the possible mechanisms involved in inflammatory processes. Rat paw edema induction by carrageenan is mediated by the release of many mediators responsible for the inflammatory reaction in two different phases [55]. The first phase occurs during the initial 2.5 h after phlogogenic agent injection and is mediated by the action of serotonin, bradykinin, and histamine on vascular permeability [56]. The second phase is accompanied by the overproduction of prostaglandins in tissue, mediated by cyclo-oxygenase (COX) [57], and can continue 5 h after injection of carrageenan [58]. Therefore, to decrease the production of these mediators, the use of natural anti-inflammatory components could represent a possible therapeutic target, and these may be used as new herbal anti-inflammatory drugs without serious side effects.

Our results revealed that VFD and the standard drug (ASL) exhibited a similar decrease in inflammatory edema volume in a dose- and time-dependent manner. The decrease in the inflammatory mediator’s release could be correlated with the richness of VFD in polyphenols and flavonoids (Table 3). In this context, it has been suggested that in addition to their antioxidant properties, flavonoids are involved in cyclooxygenase and lipoxygenase inhibition implied in arachidonic acid metabolism. Additionally, it has been concluded that the anti-inflammatory activity of VAC leaf extracts may be due to the significant reduction in pro-inflammatory cytokines (TNF-α and IL-6) by the flavonoids [45].

### 3.7. Gastroprotective Activity

Gastric ulcers are one of the most common diseases around the world. For this reason, medicinal plants can be a source of nutraceuticals and drugs with fewer side effects to treat gastric ulcer pathologies. The gastroprotective power of VFD may be related to its richness in phenolic compounds (78.53 mg GAE/g) known for their anti-ulcer and gastroprotective activities [59]. Moreover, the ability of VFD to scavenge reactive oxygen species (ROS) may contribute to the protection of gastric mucosa. In same context, it has been shown that the leaf extracts of *Vitex pubescens* exhibit a high anti-ulcer protection in rats administered with HCl/EtOH in a dose-dependent manner. Moreover, a significant increase in in vivo antioxidant enzyme activities (CAT, SOD, and GSH) was reported in gastric homogenates compared to the control group [59]. These enzymes are constituents of the endogenous antioxidant system [60] and their increment can decrease the free radical cytotoxicity of the gastrointestinal membrane [59]. Specifically, the gastroprotective properties of *Vitex agnus castus* L. extracts have been investigated in rats by other authors, who have observed that doses of 120 mg/kg showed a comparable protection to esomeprazole in gastroprotection scoring. Moreover, it has also been observed that *Vitex agnus castus* L. extracts ameliorated indomethacin-induced gastric juice acidity and pathological changes [61].

## 4. Materials and Methods

### 4.1. Reagents

Folin–Ciocalteu reagent (FC reagent), 2,2′-azinobis-3-ethylbenzothiazoline-6-sulfonic acid (ABTS), iron (II) sulfate (FeSO_4_), sulphuric acid (H_2_SO_4_), iron (III) chloride (FeCl_3_), aluminum chloride (AlCl_3_), sodium hydroxide (NaOH), ferrozine, ascorbic acid, ethylenediaminetetraacetic acid (EDTA), trichloroacetic acid (TCA),iron (II) chloride (FeCl_2_), gallic acid (98%), and quercetin (95%) were obtained from Sigma-Aldrich. The remaining chemicals were of analytical grade.

### 4.2. Plant and Decoction Preparations

*Vitex agnus-castus* L. fruits were gathered from the Gabes area (Tunisia). Identification of plant species was carried out by Professor Harzallah Skhiri Fethia (ISBM-Monastir, Tunisia). A voucher specimen was recorded in the Herbarium of the Laboratory of Bioressources: Biologie Integrative and Valorization, ISBM-Monastir, Tunisia, under number VAg20. After harvesting, the plant material was cleaned, air-dried in the shade, and then crushed using an electric grinder.

Dry fruit powder (10 g) was boiled in 1 L of distilled water for 5 min. After cooling and filtering, the decoction was frozen and lyophilized and the dry extract was designated as VFD (*Vitex* fruit decoction). The final lyophilized powder was re-dissolved in water and stored at 4 °C until phytochemical and pharmacological assays.

### 4.3. Phytochemical Studies of Vitex agnus-castus Fruit

#### 4.3.1. Total, Soluble and Insoluble Dietary Fiber

Soluble dietary fiber (SDF) and insoluble dietary fiber (IDF) were determined according to the AOAC enzymatic gravimetric method [62]. The dried *Vitex agnus-castus* fruits were incubated with three different enzymes (α-amylase, protease, and amyloglucosidase). Total dietary fiber (TDF) was calculated as the sum of SDF and IDF, as described by Chahdoura et al. (2014) [63]. The results were expressed as g per 100 g fruit (dw).

#### 4.3.2. Mineral Composition (Macro and Microelements)

Analysis was performed on dried fruits using method 930.05 of the AOAC [62] procedures. A total of 500 mg of the sample was subject to dry-ash mineralization at 550 ± 15 °C in muffle furnace for 24 h. The incineration residue was treated with HNO3 (50% *v*/*v*) and HCl (50% *v*/*v*). Thereafter, magnesium (Mg), iron (Fe), copper (Cu), and zinc (Zn) were directly measured. All the samples and standards were diluted (1/10, *v*/*v*) in the specific solutions to determine the macroelement concentrations [64]. All measurements were performed by atomic absorption spectroscopy (AAS) with an air/acetylene flame using an Analyst 200 Perkin Elmer instrument (Perkin Elmer, Waltham, MA, USA). The results were expressed in mg per 100 g fruit (dw).

#### 4.3.3. Soluble Sugars

Soluble sugars were isolated and characterized by gas chromatography [65]. After drying, the *Vitex* fruit sugars were converted into trimethylsilyl ethers in a mixture of pyridine, hexamethyldisilazane (HMDS), and trimethylchlorosilane (TMCS). Samples (1 µL) of silylated fruit extracts were analyzed using gas chromatography (GC) with a flame ionization detector (FID). The GC column was an HP-5MS capillary column (30 mm × 0.25 mm). The identification and quantification of soluble sugars were achieved according to their relative retention times in comparison with the available standards and the literature data. Values were expressed as mg per 100 g fruit (dw).

#### 4.3.4. Identification of Volatile Compounds

The volatile compounds emitted by the Tunisian *V. agnus-castus* fruits were determined for the first time by headspace solid-phase microextraction (HS-SPME) using a Supelco SPME device, Sigma Aldrich, Darmstadt, Germany (polydimethylsiloxane, PDMS, 100 μm). Sampling was achieved using the same fiber for all the analyses. After the equilibration period, the headspace was sampled for 15 min and then the fiber was inserted into the GC–MS injector.

GC–MS analyses were carried out using a Varian CP-3800 (Agilent Technologies Inc., Santa Clara, CA, USA) gas chromatograph equipped with a DB-5 capillary column (30 m × 0.25 mm; coating thickness 0.25 µm) and a Varian Saturn 2000 (Agilent Technologies Inc., Santa Clara, CA, USA) ion trap mass detector. The analyses were conducted setting the temperature of the injector and transfer line to 220 and 240 °C, respectively, while the temperature of the oven was programmed from 60 °C to 240 °C at 3 °C/min. Helium was used as a carrier gas at 1 mL/min using splitless injection. Characterization of the volatile compounds was achieved by comparing their retention times with those of pure reference compounds, their Linear Retention Indices with the series of n-hydrocarbons, and their mass spectra with those of pure substances and the MS literature data [66,67].

#### 4.3.5. Phenolic Compounds Analysis of Fruit Decoction

The total phenolic content (TPC) in *V. agnus-castus* fruit decoctions (VFD) was spectrophotometrically quantified according to the Folin–Ciocalteu procedure [68]. The outcome data were expressed as mg of gallic acid equivalents (GAE) per g of VFD.

The total flavonoid content (TFC) was determined by a colorimetric assay using AlCl_3_ [69]. The outcome data were expressed as mg of catechin equivalents (CE) per g of VFD.

The flavonol content was estimated by spectrophotometry [70] and the results were expressed as mg rutin equivalents (RE) per g of VFD. 

The condensed tannin content (CT) in the extract was determined according to Julkunen Titto (1985) [71] using the modified vanillin assay. The absorbance of the mixture was determined at 500 nm and results were expressed as mg of catechin equivalent (CE) per g of extract. Finally, the ortho-benzenediol content was estimated by gas chromatography–electron impact mass spectrometry [72] and the results were expressed as mg hydroxytyrosol equivalents (HE) per g of VFD.

### 4.4. Antioxidant Activity of Vitex agnus-castus Fruit Decoction

#### 4.4.1. DPPH Radical Scavenging Assay

The radical scavenging activity of the VFD against DPPH free radicals was determined by measuring the absorption at 515 nm [63], and BHT was used as a positive control. The IC_50_ value was thereafter deduced as the concentration of VFD providing 50% inhibition of DPPH radicals. The inhibition percentage (I%) was calculated as follows:

I% = ((A_blank_ − A_sample_)/A_blank_) × 100 (1)

where A_blank_ is the absorption (517 nm) of the control reaction and A_sample_ is the absorption (517 nm) of the tested compound.

#### 4.4.2. ABTS Radical Scavenging Assay

The antioxidant activity of VFD was also assayed using the ABTS^+^ free radical discoloration procedure [63]. The antioxidant power of VFD was expressed as the ABTS^+^ scavenging percentage, which was calculated using the following formula:

I% = ((Abs_blank_ − Abs_sample_)/Abs_blank_) × 100 (2)

where Abs_blank_ represents the absorbance (734 nm) of the control, whereas Abs_sample_ represents the absorbance (734 nm) of the sample.

#### 4.4.3. Iron-Chelating Power

The iron-chelating capacity of VFD was assessed [73]. For each assay, 50 μL of FeSO4 (2 mM) was added to 100 μL of VFD at concentrations varying between 0.03 mg/mL and 10 mg/mL. After incubation at room temperature (5 min), 100 μL of ferrozine solution (5 mM) was added to the mixtures to trigger the reaction. Finally, the test tubes were vigorously shaken and left at room temperature for 10 min. Similarly, control tubes were prepared, with the substitution of the VFD solvent and using EDTA as a positive control. The metal chelating activity (%) was calculated using the following formula:

Chelating (%) = [(Ab_C_ + Ab_B_ − Ab_S_)/Ab_C_] × 100 (3)

where Ab_C_, Ab_B_, and Ab_S_ are the control, the blank, and the sample tubes absorbances (562 nm), respectively.

#### 4.4.4. TBARS Assay

The capacity of VFD to avoid malondialdehyde generation as a result of lipid peroxidation was measured using thiobarbituric acid reactive substance (TBARS) in homogenized animal brain samples [63]. The inhibition (%) was calculated as follows:

(%) = [(Abs_control_ − Abs_sample_)/Abs_control_] × 100% (4)

where Abs_control_ and Abs_sample_ are the absorbances (532 nm) of the control and the sample, respectively.

#### 4.4.5. β-Carotene Bleaching Inhibition Assay

The capacity of VFD to avoid β-carotene bleaching inhibition was assessed [74]. The absorbance (470 nm) was determined for all samples immediately (t_0_) and at the end (t_120_) against a blank composed of an emulsion without β-carotene. The inhibition (%) was calculated as follows:

I% = (AS_120min_ − AC_120min_/AC_0min_ − AC_120min_) × 100% (5)

where AS_120min_, AC_120min_, and AC_0min_ are the absorbance of the sample at t_120_ min, the absorbance of the control at t_120_ min, and the absorbance of the control at t_0_ min, respectively. The VFD concentration (mg/mL) corresponding to 50% inhibition (EC_50_) was thereafter deduced. BHT was used as a positive standard.

#### 4.4.6. Ferric Reducing Power

The ability of VFD to reduce ferric ions (Fe^3+^) was assessed [75], and the absorbance was recorded at 700 nm. The iron power reduction capacity of the sample is associated with an increase in absorbance. BHT was used as a positive control.

#### 4.4.7. DNA Nicking Assay

The DNA protection power of VFD was analyzed using pGEM^®^-T plasmid DNA [76]. The reaction was conducted in an Eppendorf tube containing 2 μL of plasmid DNA and 5 μL of VFD (2 mg/mL). After 10 min of incubation (25 °C), 10 μL of Fenton’s reagent (3 mmol/L H_2_O_2_, 80 μM FeCl_3_, and 50 μM L-ascorbic acid) was added in the reaction mixture. Subsequently, incubation at 37 °C of the test tubes was performed for 5 min. At the end, the DNA integrity was analyzed by agarose gel electrophoresis.

### 4.5. Antimicrobial Study of Vitex agnus-castus Fruit Decoction (VFD)

#### 4.5.1. Strains

The antimicrobial susceptibility testing of VFD was performed against four Gram-positive bacteria (*Bacillus cereus* ATCC 11778, *Staphylococcus aureus* ATCC 25923, *Enterococcus epidermidis* CECT 231, and *Listeria monocytogenes* CECT933) and four Gram-negative bacteria (*Salmonella enterica* subsp. *Enterica* CECT 443, *Shigella flexneri* CECT 4804, *Pseudomonas aeruginosa* PAO1, and *Escherichia coli* ATCC 35218), while the antifungal activity of VFD was tested against four *Candida* strains (*C. tropicalis* 06-85, *C. albicans* ATCC 2019, *C. krusei* ATCC 6258, and *C. parapsilosis* ATCC 20019). CECT strains were kindly provided to Dr. Mejdi Snoussi from Professor Eulogio Valintin from the Department of Microbiology and Ecology (School of Pharmacy, University of Valencia, 46100 Burjasot, Valencia, Spain).

#### 4.5.2. Disc-Diffusion Method

The bacteria and candida broths were streaked onto MH and SB agar plates, respectively. Sterile filter discs (diameter 6 mm) were soaked in 10 µL of VFD (200 mg/mL) and thereafter aseptically distributed on the MH or SB agar media. Ampicillin (10 mg/mL; 10 µL/disc) and amphotericin B (10 mg/mL; 10 µL/disc) were used as antibacterial and antifungal positive standards, respectively [77].

After 24 h of incubation in a sterile oven (37 °C), the diameter (mm) of the growth in the inhibition zone (IZ) around each disc was measured. Each recorded value was the average of three different measurements. A low antimicrobial activity corresponds to a diameter less than 6 mm, a moderate activity corresponds to a diameter between 7 and 10 mm, a high activity corresponds to a diameter between 11 and 15 mm, and finally, a very high antimicrobial activity corresponds to a diameter exceeding 15 mm [78].

#### 4.5.3. Microdilution Method

In this study, the minimal inhibition concentrations (MICs) and the minimal bactericidal/fungicidal concentrations (MBCs/MFCs) were determined [79]. The MIC parameter was characterized as the lowest extract concentration to inhibit microorganism growth. On the other hand, the MBC/MFC values were recorded as the lowest extract concentration, which results in a clear fluid, reflecting no visible growth.

Microbial suspensions adjusted spectrophotometrically to 107 CFU/mL were used to prepare serial two-fold dilutions in nutrient broth at concentrations ranging from 0.2 to 200 mg/mL. Into each well of the plates, 95 µL of nutrient broth was added to 100 µL of a stock solution of each sample. Finally, 5 µL from each bacterial/fungal suspension was added to all wells. The first well of each plate containing only nutrient broth (195 µL) and inoculum (5 µL) was used as a negative control. Finally, plates were incubated at 37 °C for 24 h. The MBC/MIC and the MFC/MIC ratios were calculated to evaluate the antimicrobial VFD activity [80].

### 4.6. Animal Studies

Wistar rats (160–180 g) and Swiss albino mice (18–25 g) were given a standard diet and water ad libitum. They were maintained under an alternating cycle of 12 h light (L)/12 h dark (D) at 22 ± 2 °C. Before any experiment, the animals were fasted overnight with free water access. All the experimental protocols were approved by the local ethics committee of the Institute of Biotechnology (University of Monastir, Tunisia).

#### 4.6.1. Acute Oral Toxicity of *Vitex agnus-castus* Fruit Decoction (VFD)

An acute toxicity test was carried out using laboratory rats (80–100 g), which were fasted for 12 h before administration of the fruit extract. The next day, three groups (n = 6) were orally administered VFD in single doses of 100, 200, and 500 mg/kg. On the other hand, the control group was orally administered distilled water (10 mL/kg). After treatment, the animals were carefully monitored for any toxic effect during the first 4 h. Thereafter, the mortality and clinical signs such as ill health, changes in skin and fur, diarrhea, salivation, sleep, and coma were noted for 72 h and subsequently for 14 days [81]. Following these clinical observations, the LD_50_ value was deduced. On the sacrifice day, the serum was collected for clinical biochemistry measurements, such as alanine aminotransferase (ALT), aspartate aminotransferase (AST), urea, creatinine, and CRP.

#### 4.6.2. Analgesic Activity of *Vitex agnus-castus* Fruit Decoction

The acetic-acid-induced writhing method was used to evaluate the peripheral analgesic activity of VFD [82]. Thirty Swiss albino mice (18–25 g) were distributed into five groups (n = 6) 12 h prior the experiment. The first group (negative control) received distilled water at a dose of 10 mL/kg. The second group (positive control) received lysine acetylsalicylic acid (ASL) intraperitoneally (i.p.) at a dose of 200 mg/kg. The experimental group was administered three VFD doses (50, 100, and 200 mg/kg). Fifteen minutes after the administration of the decoction and reference drug (ASL), acetic acid at a dose of 10 mL/kg was intraperitoneally injected in mice to trigger the writhing reflex. Finally, the number of writhes was counted for 20 min.

#### 4.6.3. Anti-Inflammatory Activity

To evaluate the anti-inflammatory activity of VFD, carrageenan (0.05 mL, 1% *w*/*v*) was used to induce edema in the right hind paw of each rat [83]. Wistar rats (160–180 g) were randomly divided into five groups (n = 6). Before carrageenan injection (1 h), the negative control, the positive control, and the three experimental groups were administered saline solution (NaCl 9‰, 10 mL/kg), lysine acetylsalicylate (ASL) at 200 mg/kg, and VFD at different doses (50, 100, and 200 mg/kg), respectively, by intraperitoneal injection. The edema degree was evaluated by using a plethysmometer before and after carrageenan injection. The percentage inhibition of paw edema volume was calculated by the followed formula:

% inhibition = [(V_T_ − V_0_)_negative control_ − [(V_T_ − V_0_)_experimental group_/[(V_T_ − V_0_)_negative control_] × 100 (6)

where V_0_ is the basal volume and V_T_ is the edema volume after carrageenan injection at 1, 2, 3, 4, and 5 h.

#### 4.6.4. Gastroprotective Activity of *Vitex agnus-castus* Fruit Decoction (VFD)

The gastroprotective activity of VFD was studied by the induction of gastric ulcers using HCl/EtOH (40:60, *v*/*v*) [84]. Five animal groups (n = 6) were fasted 24 h prior to the test. The vehicle (NaCl 9‰) was injected intraperitoneally to the control group at a dose of 2.5 mL/kg, while the reference group received omeprazole at 30 mg/kg. Finally, the three experimental groups received VFD at doses of 50, 100, and 200 mg/kg. After 30 min, all groups were given the phologenic mixture to trigger a gastric ulcer.

After 1 h, the animals were sacrificed and their stomachs were quickly removed, opened, washed, stretched on cork plates, and photographed to analyze the gastric lesion. The extent of the observed stomach lesions was recorded (mm) and the lesion index was thereafter calculated.

### 4.7. Statistical Analysis

The obtained results are presented as the means ± SEM. A one-way ANOVA and multi-range post hoc Dunnett’s tests were used for data analyses. *p*-values less than 0.05 (*p* < 0.05) were considered statistically significant.

## 5. Conclusions

Fruits of wild *Vitex agnus-castus* L. are high in dietary fiber, as highlighted by the fact that sorbitol and arabinose are the main soluble sugars. The most abundant macroelements in these fruits are K and Ca, while Fe and Zn are the major microelements found. In addition, forty-one volatile components were detected, with 1,8-cineole comprising the majority.

This is the first study that has determined the phytochemical and pharmacological properties of fruit decoctions of *V. agnus-castus* collected in Tunisia. Our results showed that the fruit decoction was rich in polyphenols and flavonoids, which is correlated with its excellent antioxidant activities.

Therefore, it is possible to affirm that the fruit decoction of Tunisian *V. agnus-castus* demonstrated promising pharmacological activities both in vitro and in vivo studies with lab animals. Indeed, orally administered VFD did not cause any toxicity or mortality of animals up to 200 mg/kg BW and exhibited prominent analgesic and anti-inflammatory activities. Finally, pre-treatment with VFD significantly decreased gastric mucosal damage compared with the negative control group.

This work helps to validate the traditional use of *V. agnus-castus* infusions and decoctions to treat nociceptive, inflammatory, and gastric disorders and encourages researchers to further investigate the identification of pharmacological compounds from this species.

## Figures and Tables

**Table 1:** Dietary fiber (g/100 g dw) quantified by an AOAC enzymatic gravimetric method, soluble sugars (mg/100 g dw) analyzed by gas chromatography, and mineral composition (mg/100 g dw) determined by atomic absorption spectroscopy of *Vitex agnus-castus* fruits.

	Compounds	Amounts (per 100 g dw)
Dietary fiber (g/100 g dw)	Insoluble dietary fiber (IDF)	81.37 ± 2.50
	Soluble dietary fiber (SDF)	3.65 ± 0.18
	Total dietary fiber (TDF)	85.02 ± 1.37
Soluble sugars (mg/100 g dw)	Fructose	169.41 ± 0.29
	Glucose	81.21 ± 0.34
	Galactose	99.10 ± 0.06
	Arabinose	470.08 ± 0.05
	Rhamnose	41.25 ± 0.17
	Sucrose	180.49 ± 0.34
	Xylose	44.95 ± 0.02
	Mannose	272.88 ± 0.07
	Raffinose	16.42 ± 0.30
	Mannitol	68.09 ± 0.06
	Sorbitol	470.40 ± 0.28
	Maltitol	4.95 ± 0.03
	Myo-inositol	180.42 ± 0.07
Minerals (mg/100 g dw)	Cu	0.44 ± 0.01
	Mn	1.90 ± 0.02
	Zn	4.69 ± 0.05
	Fe	5.89 ± 1.26
	Ca	781.29 ± 0.59
	Mg	190.34 ± 0.79
	Na	23.28 ± 1.32
	K	1428.84 ± 8.81

**Table 2:** Volatile compounds of *Vitex agnus-castus* fruits, analyzed by GC-MS. Major component are highlighted in bold.

N°	Constituents	L.R.I ^a^	(%) ^b^
**1**	α-thujene	933	0.8
**2**	**α-pinene ^c^**	**941**	**6.2**
**3**	**sabinene**	**977**	**7.0**
**4**	β-pinene	982	1.8
**5**	myrcene	993	0.3
**6**	α-terpinene	1020	0.3
**7**	*p*-cymene	1028	0.7
**8**	**1,8-cineole**	**1034**	**30.3**
**9**	γ-terpinene	1036	0.6
**10**	*cis*-sabinene hydrate	1070	1.0
**11**	terpinolene	1090	0.2
**12**	*trans*-sabinene hydrate	1099	0.7
**13**	3-octyl acetate	1126	0.1
**14**	δ-terpineol	1172	0.3
**15**	4-terpineol	1179	0.8
**16**	α-terpineol	1191	1.0
**17**	bornylacetate	1287	0.3
**18**	δ-elemene	1340	1.1
**19**	*exo*-2-hydroxycineol acetate	1345	0.1
**20**	α-cubebene	1352	4.4
**21**	α-copaene	1377	0.1
**22**	β-bourbonene	1385	0.8
**23**	β-cubebene	1391	0.4
**24**	β-elemene	1392	0.5
**25**	α-gurjunene	1410	1.3
**26**	**β-caryophyllene**	**1419**	**9.1**
**27**	β-copaene	1430	0.5
**28**	γ-elemene	1434	0.3
**29**	*trans*-α-bergamotene	1437	0.3
**30**	aromadendrene	1440	0.2
**31**	(*Z*)-β-farnesene	1444	0.5
**32**	α-humulene	1455	0.5
**33**	**(*E*)-β-farnesene**	**1458**	**9.4**
**34**	**germacrene *D***	**1482**	**11.8**
**35**	bicyclogermacrene	1496	3.7
**36**	δ-cadinene	1524	0.3
**37**	germacrene B	1557	0.7
**38**	spathulenol	1577	0.4
**39**	caryophyllene oxide	1582	0.5
**40**	5,7-di-*epi*-α-eudesmol	1607	0.2
**41**	T-cadinol	1641	0.2
	monoterpene hydrocarbons	-	17.9
	oxygenated monoterpenes	-	34.5
	sesquiterpene hydrocarbons	-	45.9
	oxygenated sesquiterpenes	-	1.3
	non-terpene derivatives	-	0.1
**Total identified**		**-**	**99.7**

^a^ LRI: linear retention indices (HP-5 column); ^b^ %: the relative proportions of the constituents obtained by peak area normalization; ^c^ major compounds in bold.

**Table 3:** Phytochemical characterization and antioxidant activities of *Vitex agnus-castus* fruits decoction (VFD).

Phytochemical Compounds	Values (mg/g VFD)
Total phenolic content ^a^	78.53 ± 2.08
Total flavonoid content ^b^	56.52 ± 0.78
Total flavonol content ^c^	33.77 ± 0.33
Total tannin content ^d^	19.75 ± 3.51
*Ortho*-benzenediol content ^e^	30.47 ± 0.09
**Antioxidant activities**	**(EC_50_ = mg/mL VFD)**
DPPH	0.64 ± 0.11
ABTS	1.03 ± 0.02
TBARS inhibition	3.108 ± 0.074
Ferrous chelating	0.44 ± 0.12
β-carotene bleaching inhibition	0.16 ± 0.014
FRAP *	0.35 ± 0.09

Mean ± SD, n = 3. ^a^ mg GAE/g extract: mg of Gallic Acid Equivalents (GAE) per g of extract. ^b^ mg CE/g extract: mg of catechin equivalents (CE) per g of extract. ^c^ mg RE/g extract: mg of rutin equivalents (RE) per g of extract. ^d^ mg CE/g extract: mg of catechin equivalents (CE) per g of extract. ^e^ mg HE/g extract: mg of hydroxytyrosol equivalents (HE) per g of extract. EC_50_: extract concentration which corresponds to 50% of antioxidant activity. *: EC50 (mg/mL): effective concentration which corresponds to the absorbance value of 0.5.

**Table 4:** Antibacterial and antifungal activities of *Vitex agnus-castus* fruit decoction (VFD) determined by the disc diffusion and microdilution methods.

Microorganisms	Fruits Extract					Drug		
	IZ (mm)	MIC	MBC	MBC/MIC	Remarks	IZ (mm)	MIC	MBC
Bacteria Strains						Ampicillin		
*Listeria monocytogenes* CECT933	6.00 ± 0.0	3.125	50	>4	Bacteriostatic	12.66 ± 0.57	0.08	3.00
*Salmonella enterica* subsp. *Enterica* ECT443	6.00 ± 0.0	3.125	50	>4	Bacteriostatic	14.66 ± 0.57	0.2	3.00
*Staphylococcus aureus* ATCC25923	6.00 ± 0.0	3.125	50	>4	Bacteriostatic	26.66 ± 0.57	0.08	0.625
*Escherichia coli* ATCC35218	6.00 ± 0.0	3.125	50	>4	Bacteriostatic	11.67 ± 0.57	0.02	3.00
*Bacillus cereus* ATCC 11778	6.00 ± 0.0	3.125	50	>4	Bacteriostatic	26.00 ± 1.00	0.08	0.625
*Enterococcus epidermidis* CECT231	6.00 ± 0.0	3.125	50	>4	Bacteriostatic	14.33 ± 0.57	0.2	0.625
*Shigella flexneri* CECT 4804	6.00 ± 0.0	3.125	50	>4	Bacteriostatic	12.66 ± 0.57	0.2	6.00
*Pseudomonas aeruginosa* PAO1	7.00 ± 0.57	1.6	50	>4	Bacteriostatic	22.67 ± 0.57	0.1	12.00
**Yeast strains**						**Amphotericin B**		
	**IZ (mm)**	**MIC**	**MFC**	**MFC/MIC**	**Remarks**	**IZ (mm)**	**MIC**	**MFC**
*Candida parapsilosis* ATCC 20019	8.66 ± 0.66	1.6	6.25	˂4	Fungicide	12.66 ± 0.57	0.2	0.39
*Candida albicans* ATCC 2019	12.33 ± 0.32	0.4	0.8	˂4	Fungicide	12.00 ± 0.00	0.026	0.82
*Candida krusei* ATCC 6258	14.33 ± 0.33	0.4	0.8	˂4	Fungicide	10.33 ± 0.57	0.1	0.2
*Candida tropicalis* 06-85	15.66 ± 0.23	0.4	0.8	˂4	Fungicide	12.00 ± 0.00	0.42	6.75

IZ: Inhibition zone around the discs impregnated with *Vitex agnus-castus* fruits extract (10 mg/disk), ampicillin (10 mg/mL), or amphotericin B (10 mg/mL). MIC: minimal inhibitory concentration (mg/mL), MBC: minimal bactericidal concentration (mg/mL), and MFC: minimal fungicidal concentration (mg/mL). Results are expressed as the means of three replicates (mm ± SD).

**Table 5:** Liver and kidney biochemical parameters values measured in rats during the acute toxicity study on VFD.

Group	Treatment	AST (U/L)	ALT (U/L)	Urea (mmol/L)	Creatinine (µmol/L)	CRP (mg/L)
**Group I**	Control	106.5 ± 9.80	41.66 ± 3.64	7.30 ± 1.03	34.00 ± 2.39	48.33 ± 4.61
**Group II**	VFD (50 mg/kg)	111.12 ± 6.41	47.03 ± 6.22	6.62 ± 0.28	28.04 ± 1.24	47.80 ± 6.80
**Group III**	VFD (100 mg/kg)	115.00 ± 4.61	42.66 ± 5.25	4.35 ± 0.28	27.00 ± 1.15	48.5 ± 7.50
**Group IV**	VFD (200 mg/kg)	108.50 ± 8.66	44.00 ± 9.23	5.00 ± 0.15	27.00 ± 1.15	55.50 ± 5.77

ALT: alanine aminotransferase, AST: aspartate aminotransferase, CRP: C-reactive protein.

**Table 6:** Effects of *Vitex agnus-castus* fruits decoction (VFD) on acetic-acid-induced writhing in mice.

Groups	Concentration (mg/kg)	Number of Writhes	Inhibition of Writhing (%)
Negative control	-	72.80 ± 6.91	-
VFD	50	39.16 ± 5.54 ***^♪♪^	46.19
	100	25.16 ± 2.49 ***^♪^	65.43
	200	13.33 ± 1.97 ***^♪^	81.68
Reference drug (ASL)	200	18.66 ± 0.76 ***	74.35

Values are expressed as means ± SEM (n = 6). VFD: *Vitex agnus-castus* fruit decoction; ASL: acetylsalicylic acid., *** *p* < 0.001 significant versus negative control by post hoc Dunnett’s test. ^♪^ *p* < 0.05, ^♪♪^ *p* < 0.01, significant versus positive control by post hoc Dunnett’s test.

**Table 7:** Anti-inflammatory effect of VFD in the rat model of carrageenan-induced paw edema.

Sample	Dose (mg/kg)	Volume of Plantar Edema (10^−2^ mL)					Edema Inhibition (%)				
		1 h	2 h	3 h	4 h	5 h	1 h	2 h	3 h	4 h	5 h
Control	-	31.02 ± 1.08	82.58 ± 2.03	110.76 ± 2.8	118.3 ± 3.31	97.87 ± 3.05	-	-	-	-	-
VFD	50	25.03 ± 0.37 **^♪♪♪^	50.03 ± 1.59 ***^♪♪♪^	60.08 ± 1.46 ***^♪♪♪^	58.03 ± 1.57 ***^♪♪♪^	49.05 ± 4.85 ***^♪♪♪^	19.41	39.45	45.83	50.97	49.93
	100	22.03 ± 0.67 ***^♪♪^	37.06 ± 2.62 ***^♪♪♪^	38.06 ± 3.62 ***^♪♪♪^	39.01 ± 3.61 ***^♪♪^	42.03 ± 3.91 ***^♪♪♪^	29.08	55.19	65.69	67.03	57.09
	200	18.05 ± 0.81 ***^♪^	20.01 ± 1.77 ***	22.05 ± 2.41 ***	25.02 ± 2.8 ***	28.05 ± 2.49 ***^♪♪♪^	41.97	75.78	80.13	78.86	71.39
ASL (Reference drug)	200	16.42 ± 0.25 ***	17.6 ± 0.91 ***	19.58 ± 1.35 ***	21.02 ± 1.34 ***	24.16 ± 175 ***	47.04	78.68	82.32	82.24	75.30

ASL: lysine acetylsalicylic acid. Values are expressed as means ± SEM (n = 6). VFD: *Vitex agnus-castus* fruit decoction. ** *p* < 0.01, *** *p* < 0.001 significant versus negative control by post hoc Dunnett’s test. ^♪^ *p* < 0.05, ^♪♪^ *p* < 0.01, ^♪♪♪^ *p* < 0.001 significant versus positive control by post hoc Dunnett’s test.

**Table 8:** Effect of VFD on gastric ulcers induced by HCl/EtOH in rats.

Lot	Dose (mg/kg)	Ulcer Index (mm)	Inhibition (%)
Control	-	186.44 ± 8.42	-
VFD	50	126.66 ± 10.16 **^♪♪♪^	32.06
	100	56.33 ± 8.50 ***^♪♪^	69.78
	200	28.66 ± 3.05 ***^♪^	84.62
Omeprazole (reference drug)	30	10.32 ± 1.68 ***	94.46

Values are expressed as means ± SEM (n = 6). VFD: *Vitex agnus-castus* fruit decoction. ** *p* < 0.01, *** *p* < 0.001 significant versus negative control by post hoc Dunnett’s test. ^♪^ *p* < 0.05, ^♪♪^ *p* < 0.01, ^♪♪♪^ *p* < 0.001 significant versus positive control by post hoc Dunnett’s test.

## Data Availability

Not applicable.

## Sample Availability

Samples are available from the authors.
